# Bibliometric analysis of research on sarcopenia in rheumatoid arthritis: 1997–2025

**DOI:** 10.3389/fmed.2026.1756714

**Published:** 2026-08-03

**Authors:** Shuai Zhong, Shujuan Zhang, Qiuwei Peng, Jian Wang, Jiang Ping

**Affiliations:** 1 The First Clinical Medical College, Shandong University of Chinese Traditional Medicine, Jinan, China; 2 Guang'anmen Hospital China Academy of Chinese Medical Sciences, Beijing, China; 3 Rheumatology and Immunology Department, The Affiliated Hospital of Shandong University of Traditional Chinese Medicine, Jinan, China

**Keywords:** bibliometrics, visual analysis, rheumatoid arthritis, sarcopenia, VOSviewer, CiteSpace

## Abstract

**Objective:**

This study aims to utilize bibliometric techniques to examine the current state, emerging trends, research frontiers, and developmental trajectories in the field of sarcopenia in rheumatoid arthritis (RA).

**Methods:**

A comprehensive literature search was conducted in the Web of Science Core Collection (WoSCC; SCI-EXPANDED and SSCI) and Scopus databases for studies related to RA and sarcopenia from database inception to December 31, 2025. The search strategy used Boolean operators and field tags with the query: (“rheumatoid arthritis” OR “RA”) AND (“sarcopenia” OR “muscle loss” OR “muscle mass” OR “muscle wasting” OR “skeletal muscle” OR “muscular atrophy”). Only articles and reviews published in English were included. Bibliometric analyses were performed using VOSviewer (version 1.6.20) and CiteSpace (version 6.4.R1) to generate visual representations of collaboration networks, keyword co-occurrence, cluster analysis, timeline mapping, and citation burst detection.

**Results:**

A total of 433 documents (476 from WoSCC and 527 from Scopus after deduplication and screening) were included. The annual publication output increased slowly from 1996 to 2007, steadily from 2008 to 2015, and exponentially from 2016 to 2025, with 61 articles published in 2025 alone. The United States ranked first in productivity (67 publications, 5,915 citations), followed by China (63 publications, 961 citations) and Japan (58 publications, 971 citations). Osaka City General Hospital was the most productive institution (20 publications). The journal Annals of the Rheumatic Diseases published the highest number of articles (66). Keyword co-occurrence analysis identified five major clusters: rheumatoid arthritis, sarcopenia, body composition, cachexia, and skeletal muscle. Timeline and burst detection revealed a clear evolution from descriptive body composition studies (1996–2005) to inflammatory mechanisms (2006–2014), then to epidemiological characterization and non-pharmacological interventions (2015–2020), and finally to diagnostic validation and precision-oriented research (2021–2025). Emerging frontiers include multi-omics approaches (e.g., gut microbiota, cuproptosis), machine learning-based risk prediction, and targeted biologic therapies.

**Conclusion:**

Over the past three decades, research on sarcopenia in rheumatoid arthritis has expanded substantially, with rapid growth in the last five years. The field has shifted from basic description to mechanistic understanding and clinical precision. The identified research hotspots and evolutionary trends provide a valuable roadmap for future investigations into the RA-sarcopenia axis, highlighting the need for standardized screening, multidisciplinary care, and innovative translational research.

## 1 Introduction

Rheumatoid arthritis (RA) is an autoimmune disorder marked by chronic inflammation of the joint synovium, with potential systemic and multi-organ involvement. Recent research has increasingly focused on the prevention and management of RA-related complications, including interstitial lung disease (1), osteoporosis, cardiovascular disease (2), and sarcopenia (3). Sarcopenia, defined as a progressive and systemic skeletal muscle disorder characterized by the loss of muscle mass and function, predisposes individuals to adverse outcomes such as fractures, disability, and impaired mobility (4), thereby significantly affecting patient prognosis and quality of life. Epidemiological data indicate that the prevalence of sarcopenia among individuals with RA ranges from 31.0 to 54.9% (5, 6), which is substantially higher than that observed in the general population. Studies have demonstrated that sarcopenia adversely impacts physical function and disability levels in RA patients, with those exhibiting a lower Body Mass Index (BMI) experiencing diminished quality of life and elevated disability levels (7). Moreover, existing research indicates a possible bidirectional relationship between skeletal muscle and arthritis. Alterations in skeletal muscle metabolism, including enhanced amino acid catabolism and shifts in the balance between glycolysis and oxidative metabolism, have been linked to heightened rheumatoid arthritis (RA) disease activity (8). As a result, the pathogenesis and risk factors of sarcopenia in RA have increasingly become a focal point of investigation within the domain of rheumatic and immune diseases, garnering considerable attention from scholars in related disciplines.

Research on sarcopenia in RA has expanded rapidly over the past decade, driven by advances in understanding inflammatory muscle wasting, the availability of standardized diagnostic criteria (e.g., EWGSOP, AWGS), and growing recognition of its impact on patient-centered outcomes. Despite this proliferation, the literature remains fragmented across disciplines, and no comprehensive bibliometric analysis has yet mapped the intellectual structure, evolution, and emerging frontiers of this specific field. Consequently, this study utilizes bibliometric analysis to conduct an exhaustive review of pertinent literature, encompassing institutions, journals, authors, keyword co-occurrence clusters, keyword bursts, and research hotspots. By integrating a multi-omics and precision medicine perspective, this study aims to: (1) delineate the historical development and current research hotspots; (2) identify emerging topics such as the gut-muscle axis, cuproptosis, and machine learning-based risk prediction; and (3) provide an evidence-based roadmap for future investigations into the RA-sarcopenia axis.

## 2 Data and methods

### 2.1 Data collection and search strategy

To ensure a comprehensive and globally representative dataset, we systematically searched two major bibliographic databases: the Web of Science Core Collection (WoSCC; including SCI-EXPANDED and SSCI) and Scopus. These databases were selected for their extensive coverage of high-impact peer-reviewed literature in biomedical and clinical research, and their combined use is widely recommended to minimize journal coverage bias in bibliometric studies.

The search was performed on December 31, 2025, covering all records from database inception to that date. The search strategy was constructed using Boolean operators and field tags (TS for WoSCC, TITLE-ABS-KEY for Scopus) with the following query: (“rheumatoid arthritis” OR “RA”) AND (“sarcopenia” OR “muscle loss” OR “muscle mass” OR “muscle wasting” OR “skeletal muscle” OR “muscular atrophy”).

Only articles and reviews published in English were included to ensure peer-reviewed quality and international accessibility. No other restrictions (e.g., study design, sample size) were applied at the retrieval stage.

The initial search yielded 1,003 records (476 from WoSCC, 527 from Scopus). All records were exported as full bibliographic entries (including titles, abstracts, keywords, references, and citation information) in plain text format. A rigorous two-step data cleaning process was then applied:

1. Automated deduplication: CiteSpace (version 6.4. R1) was used to identify and remove duplicate records across the two databases.
2. Manual screening: Two independent reviewers (S.Z. and S.Z.) screened the titles and abstracts of the remaining records against predefined inclusion criteria. Disagreements were resolved by discussion or by consulting a senior author (J.W.). Records were excluded if they were conference abstracts, letters, editorials, news reports, or clearly irrelevant to the intersection of rheumatoid arthritis and sarcopenia.

**Figure 1:**
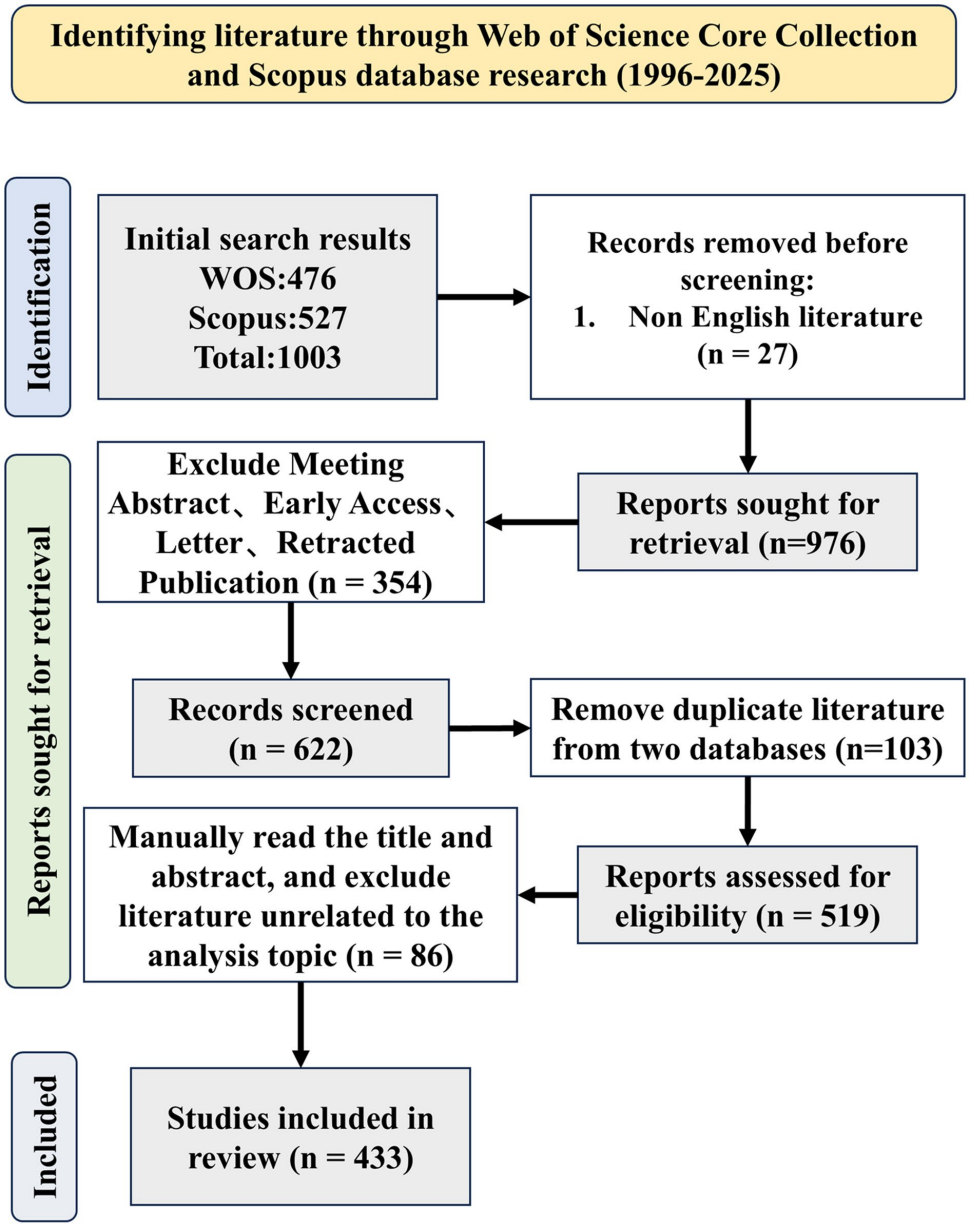
PRISMA-style flowchart detailing the identification, screening, and inclusion process of records from WoSCC and Scopus.

After deduplication and screening, a final set of 433 documents was retained for bibliometric analysis. A PRISMA-style flowchart detailing the number of records at each stage (identification, screening, inclusion) is provided as Figure 1.

### 2.2 Inclusion and exclusion criteria

The exclusion criteria encompassed literature lacking comprehensive author or institutional details, ambiguous publication years, incomplete keyword listings, duplicate entries, and materials such as conference papers, news reports, notices, and other documents not pertinent to the central theme. A total of 433 articles met the inclusion criteria and were subjected to bibliometric analysis. The detailed screening process is visualized in the PRISMA flowchart (Figure 1).

### 2.3 Analytical tools and statistical methods

Bibliometric analyses were performed using VOSviewer (version 1.6.20) and CiteSpace (version 6.4.R1).

VOSviewer was used to construct and visualize co-occurrence networks for journals, institutions, authors, and keywords. The following parameters were applied: co-occurrence type = full counting; minimum number of occurrences of a keyword = 5; minimum number of documents for an author/institution = 2; minimum number of documents for a journal = 2 (based on the exported journal data); normalization method = association strength (default). In the resulting network maps, nodes represent entities (e.g., keywords, journals), with node size proportional to the frequency of occurrence. Edge thickness indicates the strength of co-occurrence or collaboration. The VOS layout algorithm was used for spatial positioning of nodes.

**Figure 2:**
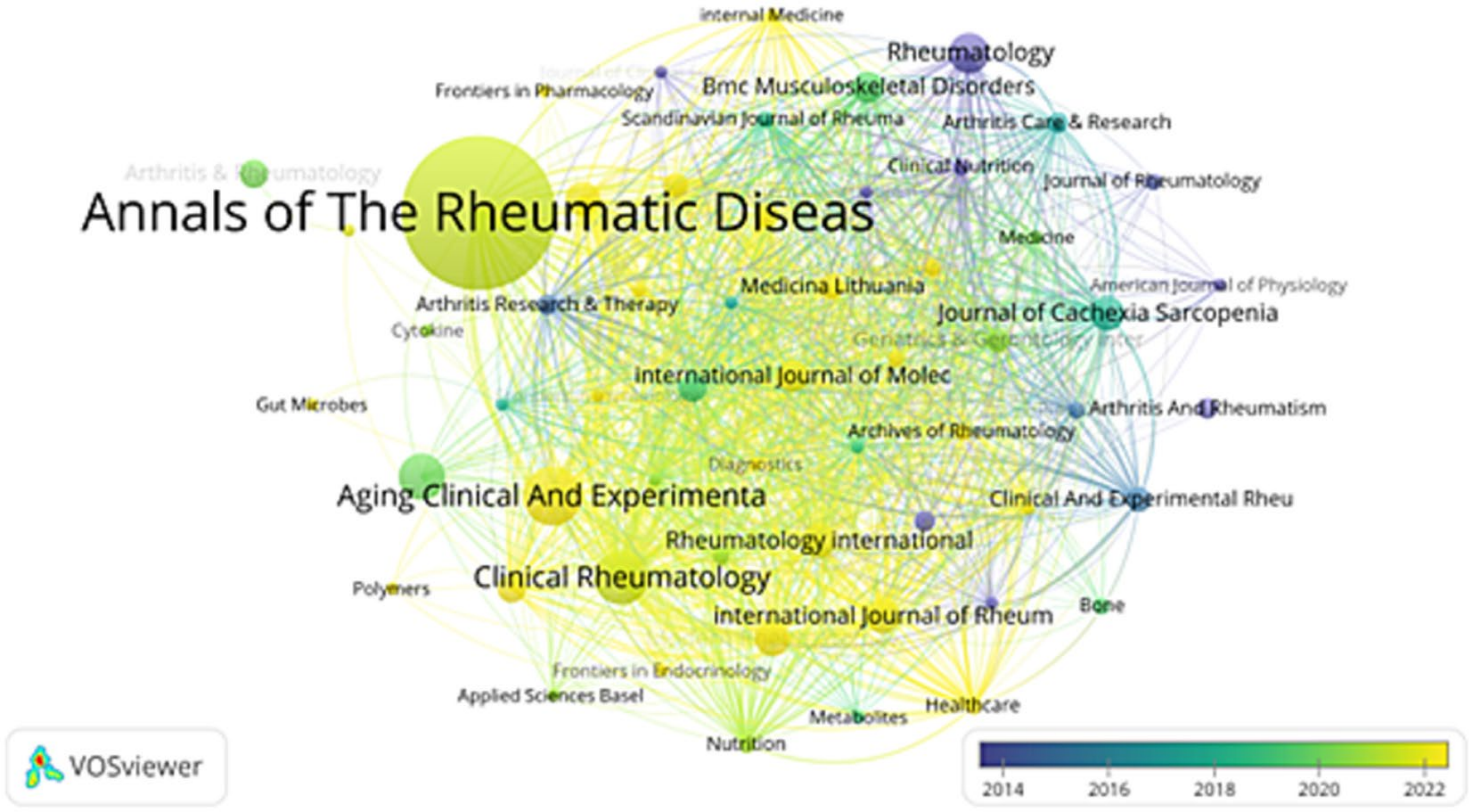
Journal co-occurrence network. Each node represents a journal; node size is proportional to the number of publications; edge thickness indicates co-citation or co-occurrence strength.

CiteSpace was used for keyword cluster analysis, timeline mapping, and burst detection. The parameters, as directly extracted from the CiteSpace run log (Figure 2B), were: time slicing = 1 year (1996–2025); selection criteria = g-index (k = 10) with LRF = 2.5, L/N = 10, LBY = 5, e = 1.0; pruning = pathfinder (without additional sliced network pruning). Cluster analysis was performed using the log-likelihood ratio (LLR) algorithm. Burst detection was configured with the default *γ* = 0.5.

### 2.4 Main observation indicators

An analysis was performed on publication volume, research institutions, journals, authors, and keywords, incorporating both co-occurrence and co-citation analyses. Additionally, keyword cluster analysis and burst detection were utilized to investigate the current research status and emerging hotspots in the study of sarcopenia in RA, with the aim of inferring potential future development trends in this field.

## 3 Results

### 3.1 Temporal trends in publication output

**Figure 3:**
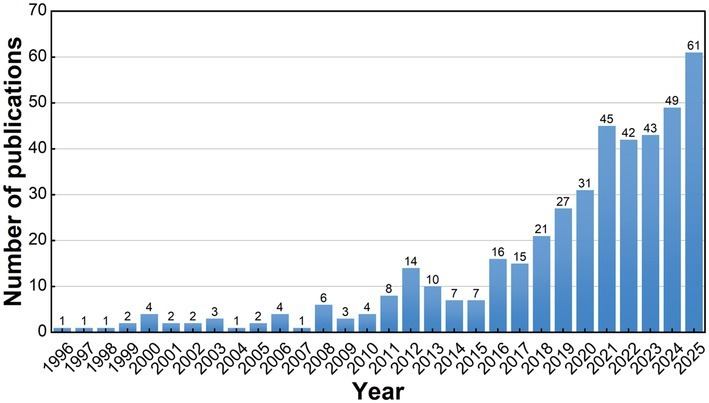
Annual publication trends of research on sarcopenia in rheumatoid arthritis from 1996 to 2025 (WoSCC + Scopus, 433 documents).

A total of 433 documents on sarcopenia in rheumatoid arthritis (RA) were retrieved from the Web of Science Core Collection and Scopus, covering the period from 1996 to 2025. Figure 3 shows the annual distribution of these publications.

From 1996 to 2007, the annual number of publications ranged from 1 to 4, with a total of 24 publications over this 12-year period. From 2008 to 2015, the annual count ranged from 3 to 14, with a total of 59 publications. From 2016 to 2025, the annual count ranged from 15 to 61, with a total of 350 publications. The highest number of publications in a single year was 61, recorded in 2025. During the five-year period from 2021 to 2025, 240 publications were issued, accounting for 55.4% of the total 433 documents.

### 3.2 Geographic distribution and collaborative networks

A total of 37 countries contributed to the 433 publications on sarcopenia in rheumatoid arthritis. Table 1 lists the top 15 countries by publication volume.

**Table 1:** Top 5 countries by publication volume in the field of sarcopenia in RA (WoSCC + Scopus, 1996–2025).

No.	Country	Publications	Total citations	Average citations per publication
1	USA	67	5,915	88.3
2	China	63	961	15.3
3	Japan	58	971	16.7
4	United Kingdom	41	1,981	48.3
5	Spain	37	604	16.3

Publications = number of documents (articles/reviews) from that country. Total citations = raw citation count. Average citations per publication = Total citations/Publications. Data: WoSCC + Scopus, 1996–2025, English articles/reviews only.

The United States ranked first with 67 publications, followed by China with 63 publications and Japan with 58 publications. The United Kingdom ranked fourth with 41 publications, Spain fifth with 37 publications, and Brazil sixth with 29 publications. Italy published 22 articles, the Netherlands 21, France 20, Germany 17, South Korea 13, Australia 12, Mexico and Turkey each 11, and Greece 10 publications. The top 15 countries collectively published 439 articles (note: this sum exceeds the total 433 due to collaborative publications counted for multiple countries).

**Figure 4:**
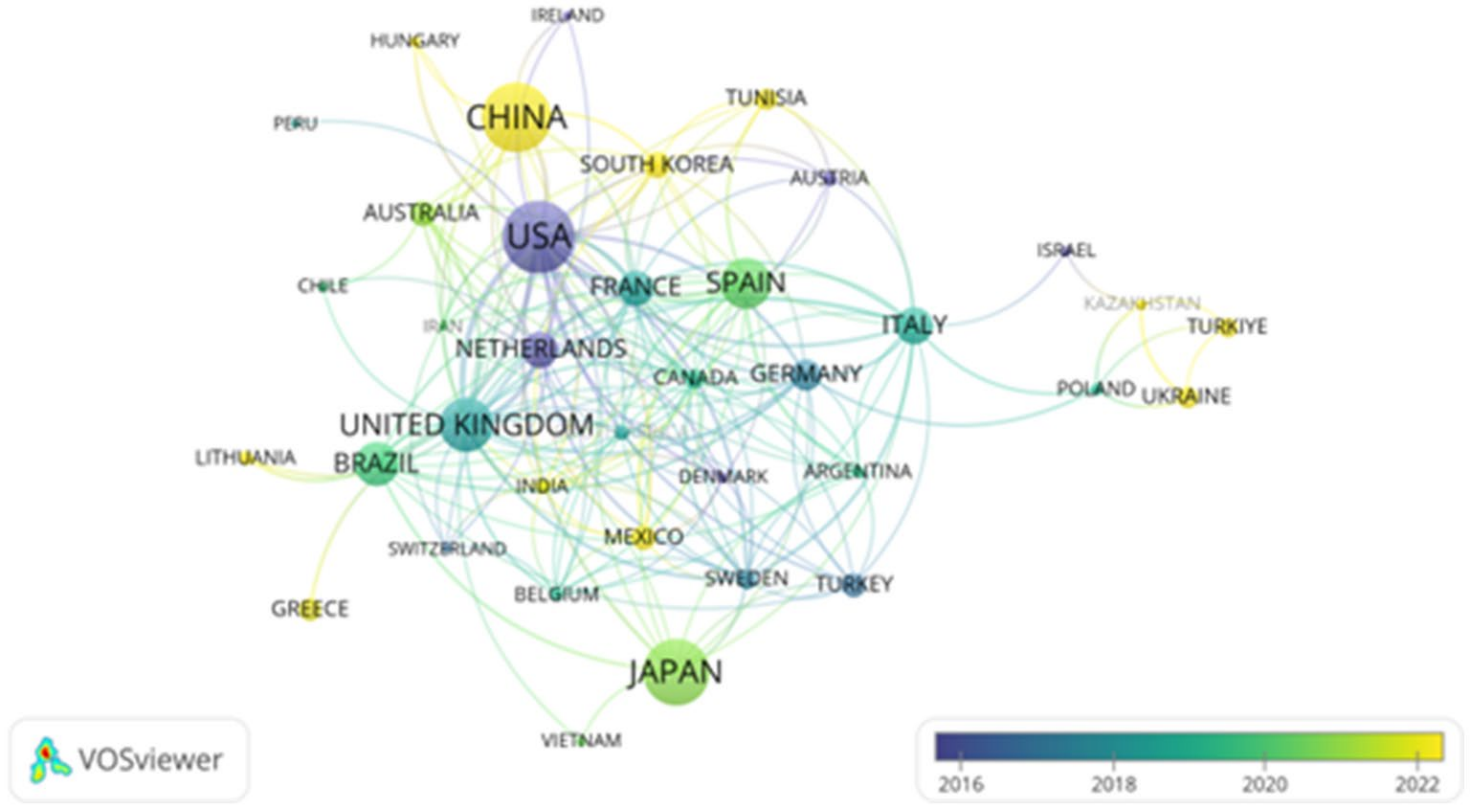
Country collaboration network. Each node represents a country; node size is proportional to the number of publications; edge thickness indicates co-authorship strength.

The country collaboration network (Figure 4) comprised 37 nodes. Link strengths and co-authorship relationships are visualized in the network map.

### 3.3 Institutional productivity and impact

A total of 91 institutions contributed to the 433 publications. Table 2 lists the top 10 institutions by publication volume.

**Table 2:** Top 10 institutions by publication volume in the field of sarcopenia in RA (WoSCC + Scopus, 1996–2025).

No.	Institution name	Norm. of pub.	Country	Total citations	Avg. pub. year
1	Osaka City Gen Hospital	20	Japan	204	2020.9
2	Osaka City University	16	Japan	216	2019.8
3	University of Birmingham	12	UK	532	2021.1
4	Maastricht University	10	Netherlands	1,001	2015.7
5	Nagoya University	10	Japan	88	2023.4
6	Sun Yat-sen University	10	China	105	2022.1
7	Hospital Clínico de Porto Alegre	7	Brazil	0.4664	2020
8	Osaka Metropolitan University	7	Japan	0.7482	2023
9	Univ Birmingham	7	UK	1.4174	2022
10	Univ Clermont Auvergne	7	France	2.0627	2020

Norm. of Pub. = Normalized number of publications (fractional counting). Total citations = Raw citation count. Avg. pub. Year = Average publication year (sum of publication years/number of documents). Higher values indicate more recent activity. Data source: WoSCC + Scopus, 1996–2025. Only articles/reviews in English. Institutions with ≥2 publications included. Ranked by Norm. of Pub.

Osaka City General Hospital (Japan) ranked first with 20 publications, followed by Osaka City University (Japan) with 16 publications. University of Birmingham (UK) ranked third with 12 publications. Four institutions each published 10 articles: Maastricht University (Netherlands), Nagoya University (Japan), and Sun Yat-sen University (China). Hospital Clinico de Porto Alegre (Brazil) and Universidade Federal do Rio Grande do Sul (Brazil) each published 9 articles. Anhui Medical University (China) and V.A. Nasonova Research Institute of Rheumatology (Russia) each published 8 articles.

**Figure 5:**
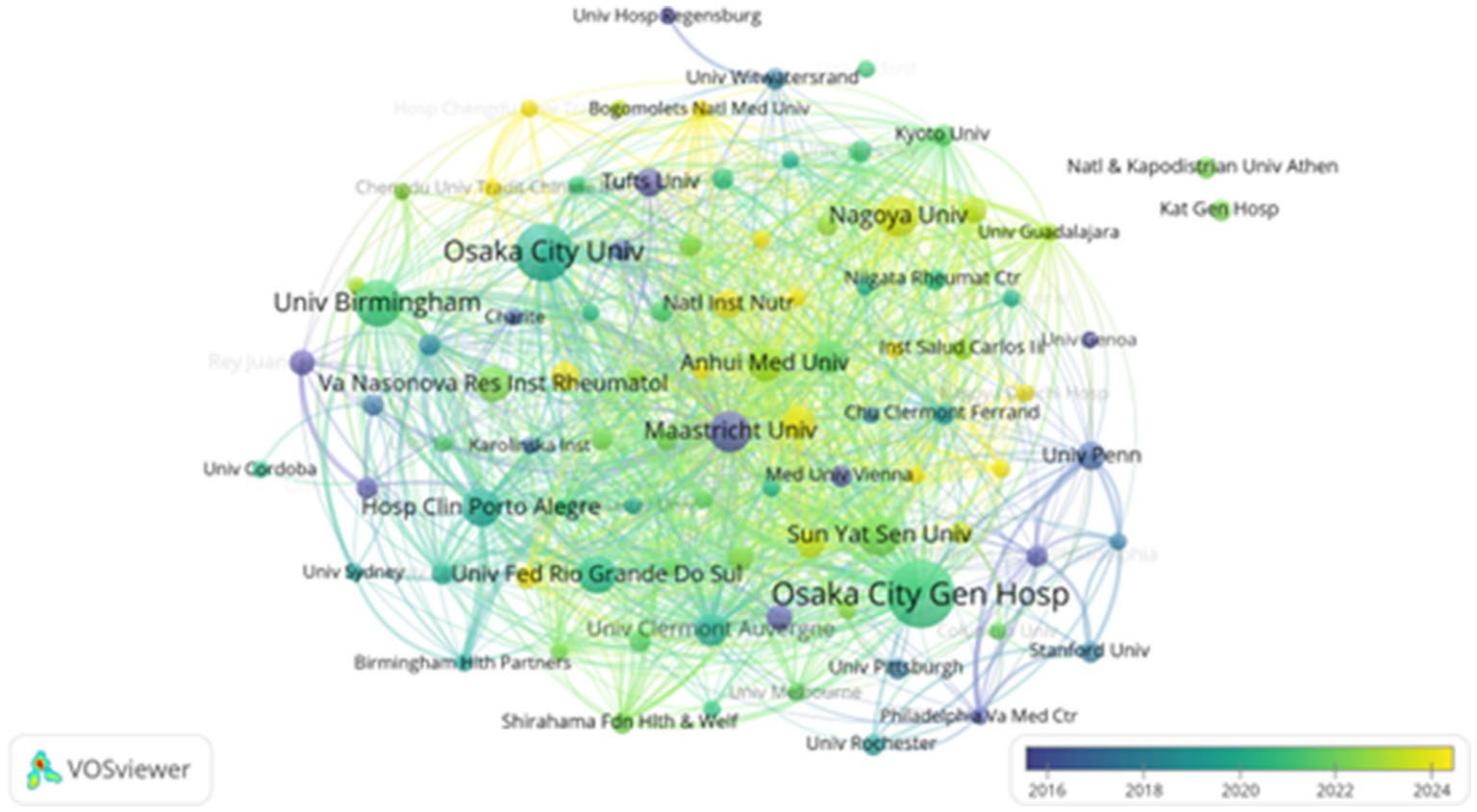
Institutional co-occurrence network. Each node represents an institution; node size is proportional to the number of publications; edge thickness indicates collaboration strength.

The top 10 institutions collectively published 112 articles, accounting for 25.9% of the total 433 publications. The institutional co-occurrence network (Figure 5) comprised 91 nodes. Link strengths and co-authorship relationships are visualized in the network map.

### 3.4 Core journals and citation influence

The 433 articles were published in 58 journals. Table 3 lists the top 5 journals by publication volume.

**Table 3:** Top 5 journals by publication volume in the field of sarcopenia in RA (WoSCC + Scopus, 1996–2025).

No.	Journal name	Norm. of pub.	Total citations	Avg. pub. year
1	Annals of the rheumatic diseases	66	38	2021.2
2	Aging clinical and experimental research	17	138	2023.0
3	Clinical rheumatology	15	279	2021.3
4	Osteoporosis international	12	4	2019.8
5	Rheumatology	10	294	2012.9

Norm. of Pub = Normalized number of publications (fractional counting). Total citations = Raw citation count. Avg. pub. Year = Average publication year (higher = more recent activity). Data source: WoSCC + Scopus, 1996–2025. Articles/reviews in English. Journals with ≥2 publications included. Ranked by Norm. of Pub.

*Annals of the Rheumatic Diseases* published the highest number of articles (66), followed by *Aging Clinical and Experimental Research* (9) and *Clinical Rheumatology* (10). *Osteoporosis International* ranked fourth with 12 articles, and *Rheumatology* ranked fifth with 10 articles. The top five journals collectively accounted for 120 articles (27.7% of the total 433).

The journal co-occurrence network (Figure 2) comprised 58 nodes. Link strengths and co-authorship relationships are visualized in the network map.

### 3.5 Leading authors and collaborative clusters

A total of 253 authors contributed to the 433 publications. Table 4 lists the top 10 authors by publication volume.

**Table 4:** Top 10 authors by publication volume in the field of sarcopenia in RA (WoSCC + Scopus, 1996–2025).

No.	Authors name	Norm. of pub	Total citations	Avg. norm. citations	Avg. pub. year
1	Mandai, K.	16	3	0.1933	2020.1
2	Toroptsova, N.	13	4	0.0998	2020.7
3	Tada, Masahiro	12	201	7.3481	2021.2
4	Demin, N.	12	4	0.0998	2020.7
5	Dobrovolskaya, O.	11	1	0.0542	2021.5
6	Xavier, Ricardo Machado	10	143	6.4913	2019.7
7	Hidaka, Noriaki	10	195	7.0232	2020.7
8	Yamada, Yutaro	9	192	6.6538	2020.3
9	Tada, M.	9	3	0.1933	2019.9
10	Nikitinskaya, O.	8	1	0.0542	2020.6

Norm. of Pub = Normalized publication count (fractional counting). Total citations = Raw citation count. Avg. norm. Citations = Average normalized citations per document. Avg. pub. Year = Average publication year (higher = more recent). Data: WoSCC + Scopus, 1996–2025. Articles/reviews in English. Authors ≥2 papers. Ranked by Norm. of Pub.

Mandai, K. was the most prolific author with 16 publications, followed by Toroptsova, N. (13 publications). Tada, Masahiro and Demin, N. each published 12 articles. Dobrovolskaya, O. published 11 articles. Xavier, Ricardo Machado and Hidaka, Noriaki each published 10 articles. Yamada, Yutaro and Tada, M. each published 9 articles. Nikitinskaya, O. published 8 articles.

**Figure 6:**
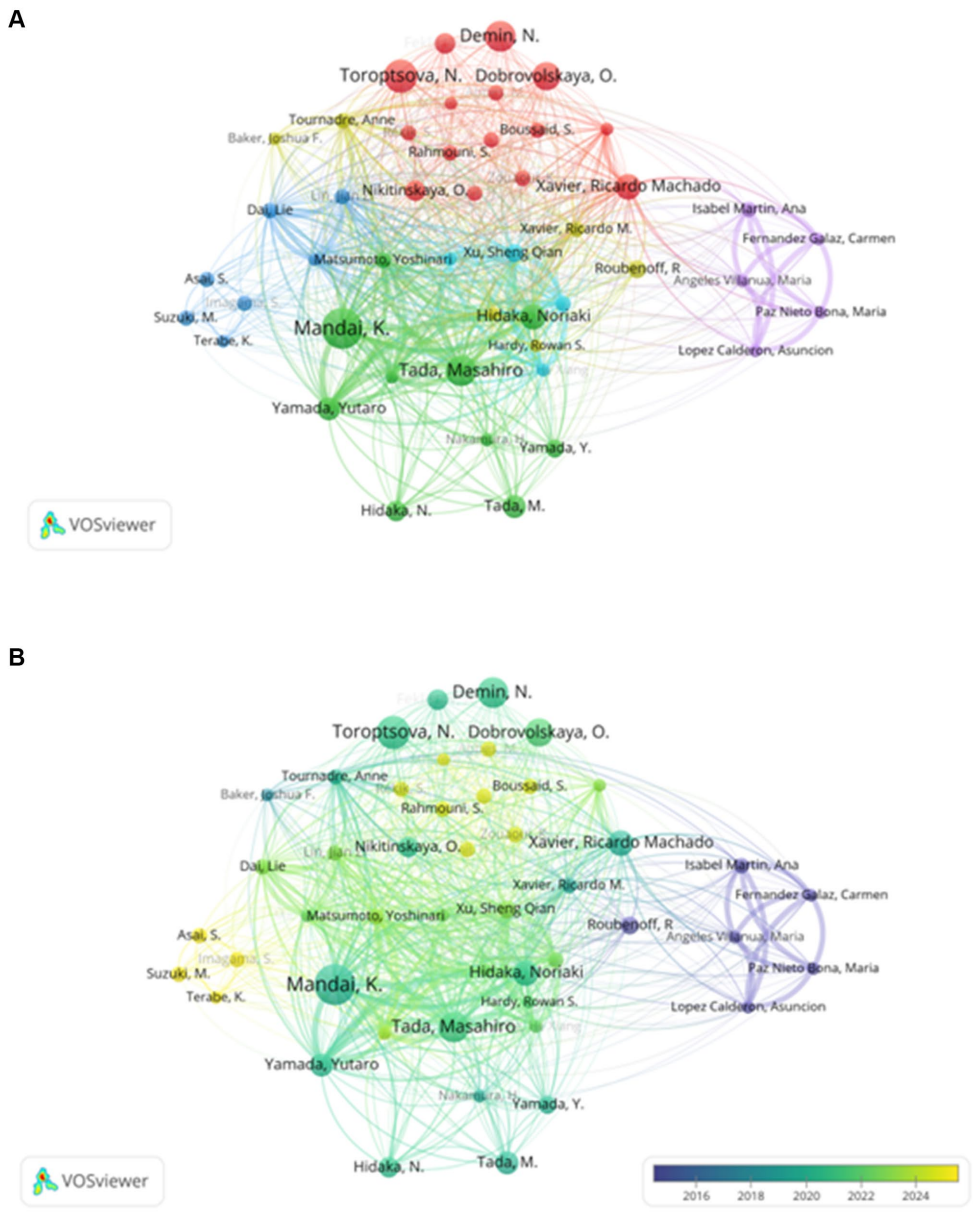
**(A)** Author collaboration network. Each node represents an author; node size is proportional to the number of publications; edge thickness indicates co-authorship strength. Colors represent different collaborative clusters. **(B)** Author co-occurrence over time. Each node represents an author; node size is proportional to the number of publications; node color indicates the average publication year (warmer colors = more recent); edges represent co-occurrence relationships.

The top 10 authors collectively published 110 articles, accounting for 25.4% of the total 433 publications. The author collaboration network (Figure 6) comprised 253 nodes. Link strengths and co-authorship relationships are visualized in the network map.

### 3.6 Keyword co-occurrence, clusters, timeline, and burst detection

**Figure 7:**
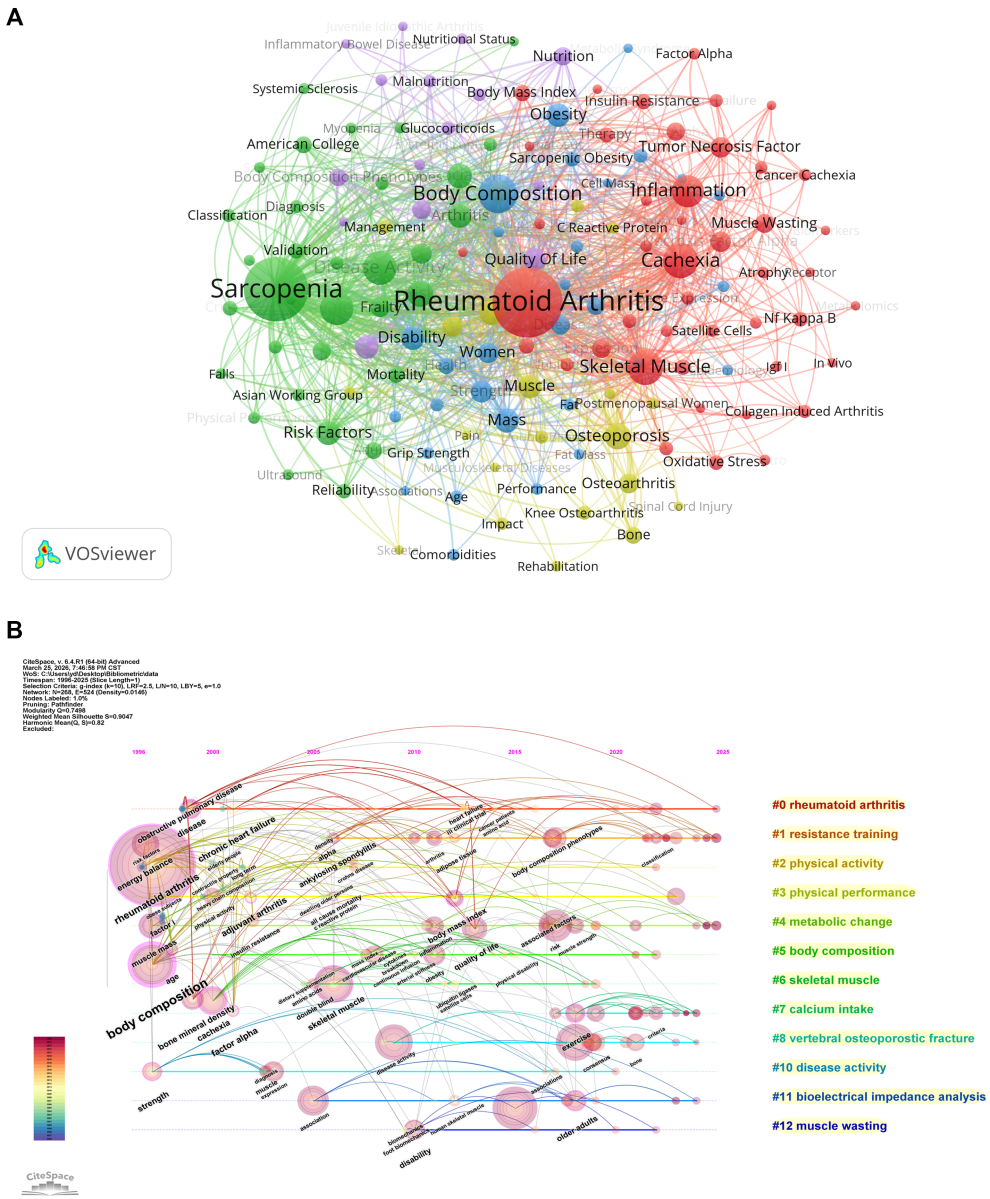

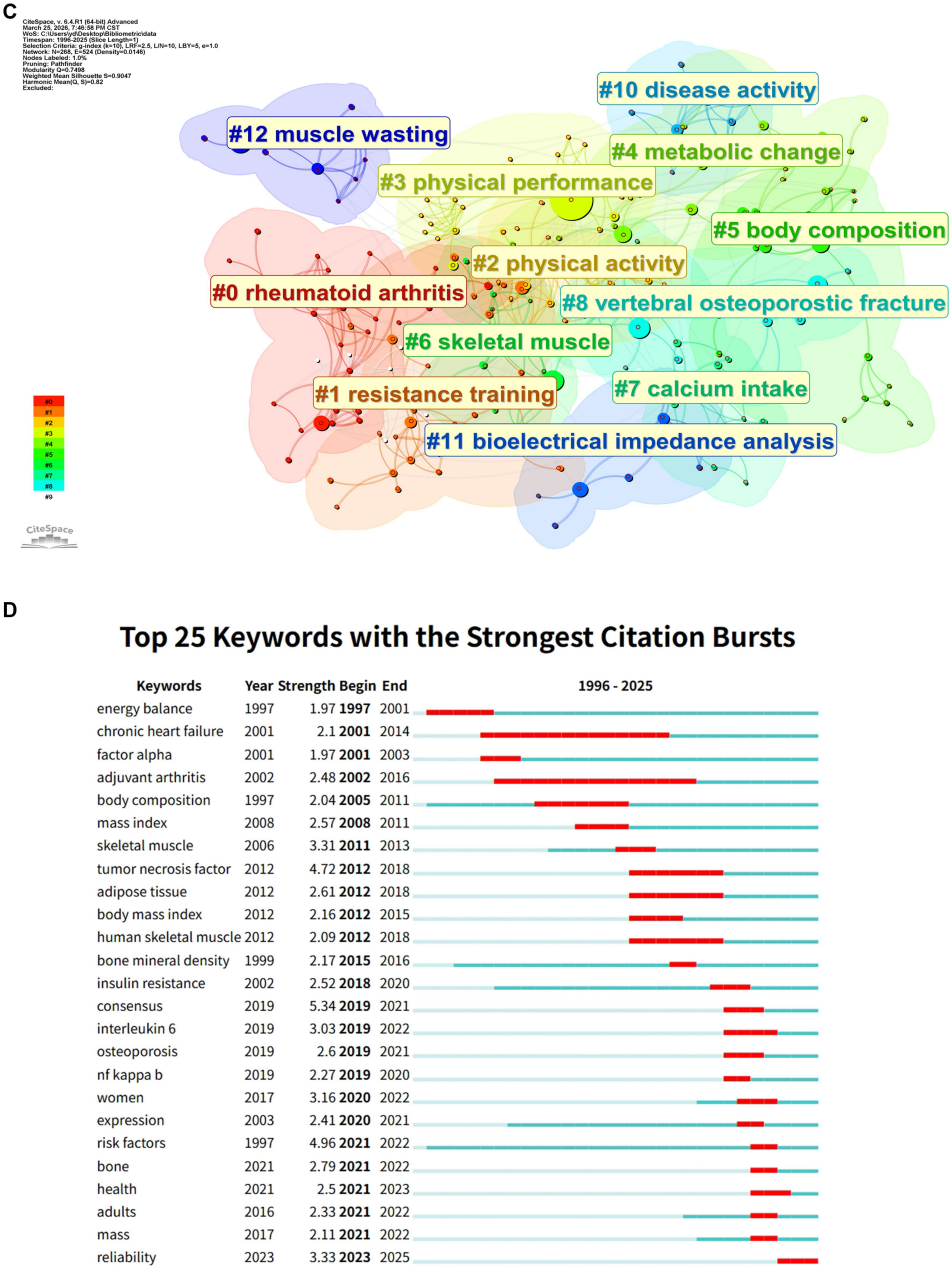
**(A)** Keyword co-occurrence map generated by VOSviewer. Node size is proportional to keyword frequency; edge thickness indicates co-occurrence strength. **(B)** Keyword cluster map generated by CiteSpace. Twelve clusters are identified and labeled. Modularity Q = 0.7498, weighted mean silhouette S = 0.9047. **(C)** Keyword timeline view showing the temporal evolution of clusters from 1996 to 2025. Each horizontal line represents a cluster; keywords are placed according to their first appearance year. **(D)** Keyword burst detection map. Red bars indicate periods of sharp increase in citation frequency for each keyword. The strongest burst is “Consensus” (strength 5.34, 2019–2021).

Keywords encapsulate the core content of scholarly articles. A keyword co-occurrence analysis was performed using VOSviewer (Figure 7A). The resulting network comprised 107 nodes and 4,456 links. The five most frequent keywords were: “Rheumatoid Arthritis” (247 occurrences) “Sarcopenia” (204) “Body Composition” (75) “Cachexia” (62) and “Skeletal Muscle” (61). Cluster analysis was conducted using CiteSpace (version 6.4.R1) with the log-likelihood ratio (LLR) algorithm time slicing of 1 year (1996–2025) selection criteria g-index (k = 10) and pathfinder pruning. The analysis yielded 12 distinct clusters (Figure 7B). The modularity Q was 0.7498 and the weighted mean silhouette S was 0.9047 indicating well-defined themes and high cluster homogeneity. The identified clusters were: #0 rheumatoid arthritis #1 resistance training #2 physical activity #3 physical performance #4 metabolic change #5 body composition #6 skeletal muscle #7 calcium intake #8 vertebral osteoporotic fracture #10 disease activity #11 bioelectrical impedance analysis and #12 muscle wasting. The timeline view (Figure 7C) illustrates the temporal evolution of these clusters showing a shift from basic body composition research (late 1990s) toward mechanistic pathways (2005–2014) then to prevalence and diagnostic consensus (2015–2020) and finally to clinical validation and precision management (2021–2025). Burst detection (Figure 7D) identified keywords with a sharp increase in citation frequency. The strongest burst was “Consensus” (strength 5.342019-2021) followed by “Risk factors” (strength 4.962021-2022). Other significant bursts included “Tumor necrosis factor” (strength 4.722012-2018) “Interleukin 6” (strength 3.032019–2022) and “Reliability” (strength 3.332023-2025). These results collectively map the intellectual structure and evolving research hotspots in the field of sarcopenia in rheumatoid arthritis (see Table 5).

**Table 5:** Top 10 keywords by occurrence frequency in the field of sarcopenia in RA (WoSCC + Scopus, 1996–2025).

No.	Key word	Count
1	Rheumatoid arthritis	247
2	Sarcopenia	204
3	Body composition	75
4	Cachexia	62
5	Skeletal muscle	61
6	Prevalence	57
7	Disease activity	56
8	Inflammation	52
9	Risk	36
10	Osteoporosis	35

Count = raw frequency of the keyword in the dataset (minimum 5 occurrences). Data source: WoSCC + Scopus, 1996–2025; articles and reviews in English only.

## 4 Discussion

### 4.1 General research overview

Our bibliometric analysis retrieved 433 documents on sarcopenia in RA from the Web of Science Core Collection and Scopus, covering the period from 1996 to 2025. The annual publication trend (Figure 3) reveals a clear three-phase growth pattern.

#### 4.1.1 Phase 1 (1996–2007)

A total of 24 articles were published, with annual output ranging from 1 to 4 articles. This phase represents the germination period of the field, during which research interest remained extremely low.

#### 4.1.2 Phase 2 (2008–2015)

A total of 59 articles were published. Annual output first reached double digits during this period, peaking at 14 articles in 2012. This steady growth coincided with the publication of standardized sarcopenia definitions by international working groups (e.g., EWGSOP), although the present bibliometric analysis does not establish causality.

#### 4.1.3 Phase 3 (2016–2025)

A total of 350 articles were published, accounting for 80.8% of all included documents. Annual output increased from 16 articles in 2016 to a peak of 61 articles in 2025. Notably, 240 of these 350 articles (68.6%) were published between 2021 and 2025, indicating that the majority of research activity has occurred in the most recent five-year window.

Overall, the field of sarcopenia in RA has experienced exponential growth over the past three decades, with more than half of all publications appearing since 2021. This temporal distribution underscores that the RA-sarcopenia axis has become an increasingly active research domain.

### 4.2 Geographical and institutional contributions

The country-level analysis (Figure 4; Table 2) shows that 37 countries contributed to the 433 publications. The United States ranked first with 67 publications and 5,915 citations, followed by China (63 publications, 961 citations) and Japan (58 publications, 971 citations). Although China and Japan are close to the US in publication volume, their total citations are substantially lower. European countries demonstrated high citation efficiency: the United Kingdom (41 publications, 1,981 citations), the Netherlands (21 publications, 1,607 citations), and Italy (22 publications, 1,576 citations) each achieved average citations per publication exceeding 70, compared to approximately 15 for China and 17 for Japan.

At the institutional level (Table 2; Figure 5), 91 institutions participated. Osaka City General Hospital (Japan) was the most productive institution (20 publications), followed by Osaka City University (Japan, 16 publications) and the University of Birmingham (UK, 12 publications). Maastricht University (Netherlands) published only 10 articles but accumulated 1,001 citations (average 100.1 per article), the highest among the top 10 institutions. Similarly, Tufts University (USA) had 6 publications with 901 citations (average 150.2). In contrast, Japanese institutions – despite high productivity – showed lower average citations (e.g., Osaka City General Hospital: 204 citations from 20 articles, average 10.2). The institutional co-authorship network (Figure 2) revealed dense collaboration within geographic clusters, including the “Osaka cluster” in Japan, a European network centered on Maastricht and Birmingham, and an emerging Chinese cluster involving Sun Yat-sen and Anhui Medical Universities.

These findings indicate a dichotomy between volume-driven productivity (predominantly in East Asia) and high-impact foundational research (predominantly in Europe and the United States). The temporal overlay of the collaboration network (Figure 4) further suggests that research momentum has recently shifted toward emerging economies, with China, Brazil, and Turkey showing increased activity since 2020.

### 4.3 Evolution of research hotspots (based on keyword clusters and timeline)

The keyword timeline view (Figure 7C) and cluster analysis (Figure 7B) allow a chronological mapping of thematic shifts in the RA-sarcopenia field from 1996 to 2025. Based on the temporal distribution of keywords, the evolution can be divided into four consecutive phases.

#### 4.3.1 Phase 1 (1996–2005)

The earliest research focused on basic body composition and musculoskeletal parameters. Keywords such as “body composition” (first appeared 1997), “muscle mass” (1997), “bone mineral density” (1999), and “risk factors” (1997) dominated this period. These terms belong to clusters #5 (body composition) and #6 (skeletal muscle), indicating an initial descriptive approach to physical changes in RA without deep mechanistic exploration.

#### 4.3.2 Phase 2 (2006–2014)

A clear transition toward molecular and inflammatory mechanisms occurred. Keywords including “skeletal muscle” (2006), “necrosis factor alpha” (2008), “inflammation” (2011), and “tumor necrosis factor” (2012) emerged. Clusters #4 (metabolic change) and #12 (muscle wasting) became prominent. Simultaneously, clinical outcome terms such as “disability” (2010) and “quality of life” (2013) appeared, linking biological pathways to patient-relevant endpoints.

#### 4.3.3 Phase 3 (2015–2020)

This period witnessed the formal adoption of “sarcopenia” as a key concept (first appeared 2015). High-frequency keywords included “prevalence” (2018), “consensus” (2019), “older adults” (2018), and “frailty” (2020). Clusters #2 (physical activity), #1 (resistance training), and #3 (physical performance) expanded, reflecting a shift toward epidemiological characterization and non-pharmacological interventions. The burst detection identified “consensus” as the strongest burst (strength 5.34, 2019–2021), underscoring the field’s effort to standardize diagnostic criteria.

#### 4.3.4 Phase 4 (2021–2025)

The most recent phase is characterized by clinical validation and precision management. Keywords such as “validation” (2023), “criteria” (2022), “management” (2022), “Sarc-F” (2025), “handgrip strength” (2025), and “biological DMARD” (2025) entered the network. Cluster #10 (disease activity) and #11 (bioelectrical impedance analysis) remained active. Emerging low-frequency terms – “gut microbiota” (2023), “Mendelian randomization” (2024), “machine learning” (not shown in top list but present in raw data), and “cuproptosis” (2025) – indicate that multi-omics and computational approaches are beginning to be applied to the RA-sarcopenia axis.

Overall, the keyword evolution shows a clear progression from descriptive body composition studies (phase 1) to inflammatory mechanisms (phase 2), then to epidemiology and non-drug interventions (phase 3), and finally to diagnostic validation and precision-oriented research (phase 4). This thematic sequence is consistent with the overall publication growth pattern described in Section 4.1.

### 4.4 Limitations and summary

This study has several limitations that should be acknowledged. First, although we searched both the Web of Science Core Collection and Scopus to minimize journal coverage bias, other important databases (e.g., PubMed, Embase) were not included. The exclusion of these databases may have led to the omission of relevant publications, particularly those not indexed in WoSCC or Scopus. Second, only English-language articles and reviews were included. Non-English publications, which might contain valuable regional data, were excluded, potentially introducing language bias. Third, bibliometric analyses are inherently descriptive and do not assess the methodological quality, clinical validity, or risk of bias of individual studies. Fourth, name variations of authors (e.g., “Tada, Masahiro” vs. “Tada, M.”) and institutions (e.g., “Osaka City Gen Hosp” vs. “Osaka City General Hospital”) may have resulted in incomplete merging or duplicate counting, despite manual curation efforts. Fifth, citation-based indicators, such as burst detection, reflect past attention rather than future impact; emerging topics with low current citation counts may be underrepresented. Finally, the total number of publications in this field, although growing, remains relatively modest, which may limit the robustness of some network metrics. In summary, this bibliometric analysis of 433 documents from WoSCC and Scopus (1996–2025) provides a comprehensive mapping of the research landscape on sarcopenia in rheumatoid arthritis. The field has experienced exponential growth, particularly in the last five years, with the United States, China, and Japan leading in productivity, while European centers demonstrate high citation impact. Keyword evolution reveals a clear progression from descriptive body composition studies (phase 1) to inflammatory mechanisms (phase 2), then to epidemiology and non-pharmacological interventions (phase 3), and finally to diagnostic validation and precision-oriented research (phase 4). Emerging frontiers include multi-omics (e.g., gut microbiota, cuproptosis), machine learning-based risk prediction, and targeted biologic therapies. Despite the limitations noted above, this study offers a valuable roadmap for clinicians and researchers, highlighting the need for standardized screening, multidisciplinary care, and innovative mechanistic investigations to improve outcomes in RA patients with sarcopenia.

## 5 Supplementary note: a narrative overview of key mechanistic and clinical advances in RA-sarcopenia research

The following narrative overview is provided as supplementary context to complement the bibliometric findings, summarizing key clinical and mechanistic studies cited in the literature. RA sarcopenia research has undergone a complete process: from raising the question, to clinical verification, then to mechanistic exploration, and finally discussing intervention strategies. In the late 1990s to early 2000s, sarcopenia was still studied as an independent geriatric syndrome. Although the role of chronic inflammatory diseases in its pathogenesis was mentioned, it was not fully recognized. Early research primarily focused on the definition, diagnostic criteria, and prevalence of sarcopenia in the elderly (11, 12). After 2000, with a deeper understanding of the “inflammaging” mechanism and developments in RA-related cachexia research, scholars began focusing on the impact of chronic inflammation on muscle mass and function. In 2006, Roubenoff et al. explicitly proposed the “role of inflammatory cytokines in age-related muscle loss,” laying the theoretical foundation for RA sarcopenia research (13). In 2008, Pippa et al. first explicitly raised the question “Does RA really have sarcopenia?” in *Arthritis and Rheumatism*, formally initiating exploration in this field. From 2008–2014, research mainly focused on confirming the presence and clinical significance of sarcopenia in RA patients. Mechanistic exploration began but was largely confined to inflammation-related pathways. In 2010, Beenakker et al., through a systematic review, indicated that decreased handgrip strength in RA patients was closely related to chronic inflammation, suggesting inflammation as a key trigger for sarcopenia (14). In 2012, Teixeira et al. further systematically elaborated the molecular mechanisms of sarcopenia in rheumatic diseases in *Revista Brasileira de Reumatologia*, including protein degradation pathways, apoptosis, and impaired muscle regeneration (15), laying the groundwork for subsequent mechanistic studies.

From 2015 to 2019, with the gradual standardization of sarcopenia diagnostic criteria (e.g., EWGSOP, AWGS) (10, 16), the research focus shifted to prevalence and risk factors. Multiple cross-sectional studies (e.g., by Torii, Mochizuki, Tada) reported a sarcopenia prevalence of 25–40% in RA patients, significantly higher than in healthy peers (9, 17, 18). Risk factors included advanced age, long disease duration, high disease activity (DAS28), elevated inflammatory markers (CRP, MMP-3) (19), and glucocorticoid use. Furthermore, the use of biological DMARDs was found potentially associated with a reduced risk of sarcopenia, highlighting the importance of inflammation control for muscle health (20).

From 2020–2023, research exhibited multidimensional and interdisciplinary characteristics. On one hand, epidemiological studies expanded sample sizes, covered diverse populations (21–23), and incorporated updated diagnostic criteria like EWGSOP2, improving the sensitivity of sarcopenia identification. On the other hand, genetics and molecular biology methods were introduced; for instance, Mendelian randomization studies revealed a potential causal relationship between RA and sarcopenia and identified shared genes and signaling pathways (e.g., BMP, cGAS-STING) (24). Concurrently, treatment-related research emerged, evaluating the effects of biologics and targeted drugs (e.g., JAK inhibitors, TNF-*α* inhibitors) on muscle mass and function (25). Non-pharmacological interventions like nutritional support and exercise training (including resistance and aerobic exercise) were also confirmed to have positive effects on improving RA sarcopenia (26–28). Comorbidity studies gained prominence, exploring associations between osteoporosis and RA sarcopenia, and the emergence of RA with sarcopenic obesity (29, 30).

In recent years, with advancements in bioinformatics, RA sarcopenia research has progressed towards greater precision and personalization, with increasingly detailed and rich mechanistic studies. Machine learning models and risk prediction nomograms have been developed for early identification of high-risk patients (31, 32). Novel mechanisms such as the gut microbiota-muscle axis (33), cuproptosis (34), and metabolic reprogramming (35) have become research hotspots. In summary, research in the field of RA sarcopenia is becoming increasingly broad and popular. However, relatively underexplored areas remain, such as pharmacological intervention strategies, and there is still a lack of large-scale epidemiological studies confirming the incidence of RA sarcopenia.
